# Operative Therapie der Arthrofibrose des Kniegelenks

**DOI:** 10.1007/s00113-022-01242-4

**Published:** 2022-10-17

**Authors:** Michael Jagodzinski, Philipp Traut

**Affiliations:** 1 Agaplesion Ev. Klinikum Schaumburg, Zum Schaumburger Klinikum 1, 31683 Obernkirchen, Deutschland; 2 Praxis für orthopädische Beratung und Begutachtung, Herforder Str. 45, 32545 Bad Oeynhausen, Deutschland

**Keywords:** Totalendoprothese, Bewegungseinschränkung, Arthroskopie, Arthrolyse, Komplikation, Total knee arthroplasty, decreased range of motion, Arthroscopy, Arthrolysis, Complication

## Abstract

Die Arthrofibrose des Kniegelenks ist eine schwerwiegende Komplikation nach Trauma und Operation, da die Funktion des Gelenks häufig dauerhaft beeinträchtigt wird. Es werden nach wie vor frühzeitige Mobilisierungstechniken und die Anästhesie eingesetzt, ohne dass die zugrunde liegenden Prozesse ausreichend aufgeklärt wurden. Während die Frühphase der Arthrofibrose gut auf konservative Maßnahmen zur Schmerzreduktion und zur Wundheilungsregulation anspricht, ist in der Spätphase häufig straffes kollagenes Narbengewebe vorhanden, das die Beweglichkeit dauerhaft einschränkt. In dieser Phase ist eine Verbesserung der Beweglichkeit ohne chirurgische Maßnahmen in der Mehrzahl der Fälle aussichtslos. Bei einer chirurgischen Therapie sollte zwischen der lokalisierten (zumeist sekundären) Arthrofibrose (z. B. Kreuzbandoperation) und einer generalisierten Arthrofibrose (primär, in der Mehrzahl der Fälle nach einer Knietotalendoprothese [Knie-TEP]) unterschieden und die Behandlung entsprechend geplant werden. Begleitende pathologische Veränderungen (Transplantatposition, Instabilität der TEP, Implantatverschleiß, „Low-grade“-Infektion, patellofemorale Instabilität oder „maltracking“, Patella baja) müssen bei der Behandlung berücksichtigt werden. Eine multimodale Begleitbehandlung (Physiotherapie, Schmerztherapie, Psychosomatik) ist zur Sicherung des Behandlungserfolgs notwendig.

## Abstract (en)

Arthrofibrosis of the knee joint is a severe complication following trauma and surgical procedures, which often results in long-term impairment of joint function. Early mobilization techniques and anesthesia are still employed without sufficient clarification of the underlying processes. While the early stages of arthrofibrosis can be successfully treated with conservative measures for pain reduction and wound healing, in the late stage tense collagenous scar tissue is frequently present that permanently limits joint mobility. In this stage an improvement of joint mobility has no chance of success without a surgical intervention. In surgical treatment a differentiation should be made between localized (mostly secondary) arthrofibrosis (e.g. cruciate ligament surgery) and generalized arthrofibrosis (in the majority of cases primarily after total knee arthroplasty) and the treatment planned accordingly. Comorbid pathological alterations (transplant position, instability of the total knee endoprosthesis, implant attrition, low-grade infection, patellofemoral instability or maltracking, patella baja) must be taken into consideration in the treatment. A multimodal accompanying treatment including physiotherapy, pain therapy and psychosomatics is necessary to ensure successful treatment.

## Einführung zum Thema

Eine Arthrofibrose nach Kreuzband- [1] oder Knorpelrekonstruktion [2] oder nach der Implantation eines partiellen oder eines totalen Kniegelenkersatzes [3] stellt eine schwerwiegende Komplikation dar, da die Bewegungseinschränkung häufig persistiert und eine hohe Rezidivquote nach einer Narkosemobilisierung angegeben wird [4].

Durch ein verbessertes Verständnis der inflammatorischen Prozesse, die an der Entwicklung einer Arthrofibrose beteiligt sind („alpha-smooth muscle actin“, α‑SMA [5]; „transforming growth factor beta“, TGF‑β; „xylosyltransferase 1“, XT‑1 [6], und XT‑2 [6]), haben sich die Behandlungsoptionen mittlerweile verbessert. Hauptaugenmerk liegt auf der frühzeitigen Diagnose sowie der Reduktion der Inflammation und Wachstumsfaktoren [5].

Die Arthrofibrose wird nach Totalendoprothesenimplantation mit einer Inzidenz von 1–13 % [7] angegeben. Die Gelenkfunktion wird langfristig beeinträchtigt [7].

Bei später Diagnose oder inadäquater Therapie kommt es jedoch häufig zur Ausbildung eines straffen Narbengewebes, das antiinflammatorischer Therapie nicht mehr zugänglich ist. Für diese Fälle werden eine arthroskopische Arthrolyse, ein Versatz der Tuberositas oder die Durchtrennung der Quadrizepssehne oder ein Wechsel der Totalendoprothese (TEP) mit Resektion der Narben als probate Optionen angegeben und mit guten Ergebnissen berichtet [8]. Ein systematischer Vergleich dieser Operationstechniken mit den jeweiligen Ergebnissen fehlt bisher.

Ziel einer jeden Reoperation muss es sein, die möglichen Ursachen der Bewegungseinschränkung zu analysieren, um anschließend eine kausale Therapie durchzuführen. In dieser Hinsicht ist bemerkenswert, dass die Arthrofibrose nach der Implantation einer Kniegelenk-TEP zusätzlich zu anderen pathologischen Veränderungen auftreten kann (Abrieb, Instabilität, Mischtyp; [9]).

## Diagnostische Arthroskopie

**Figure 1:**
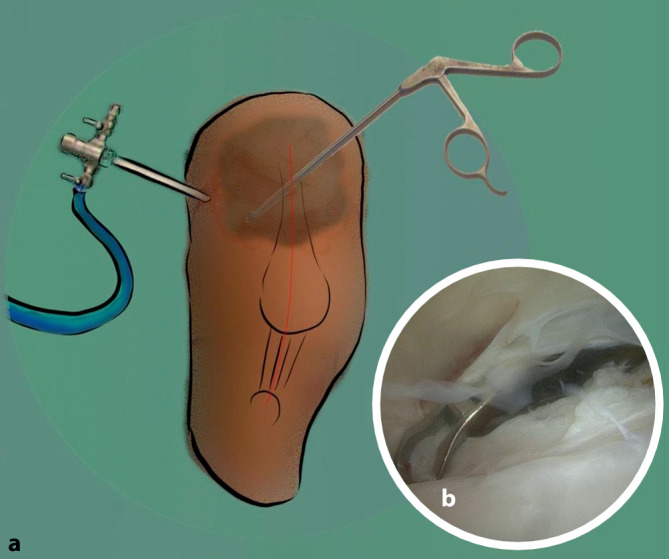

Während die diagnostische Arthroskopie bei Meniskus- und Kreuzbandverletzungen in der heutigen Zeit obsolet ist [10], kann eine diagnostische Arthroskopie bei unklarer pathologischer Ursache einer Bewegungseinschränkung sinnvoll sein. Hier können zeitgleich Proben zur mikrobiologischen und zur histologischen Aufarbeitung analysiert werden [9]. Bei implantierter Endoprothese ist eine Entnahme über den oft sehr engen oberen Recessus mit einem reduzierten Risiko für die Beschädigung des Implantats verbunden. Das Arthroskop wird von lateral in den oberen Recessus eingebracht. Die Proben können mithilfe der Arthroskopie sicher am Übergang der Synovia zur Gelenkkapsel unter Sicht aus den pathologischen Arealen entnommen werden (Abb. 1): Die Biopsie zur histologischen Absicherung der Diagnose erfolgt arthroskopisch unter Sicht aus den betroffenen Arealen des Gelenks. Mindestens drei 3–5 mm durchmessende Proben sollten aus repräsentativen Bereichen nahe der Endoprothese und in Entfernung zum Implantat entnommen werden. Bei pathologischen Knochenveränderungen sollten zusätzlich knöcherne Proben entnommen werden [9]. Im Fall einer implantierter Knie-TEP bietet sich eine Entnahme über die alte zentrale Narbe an. Gleichzeitig können die Position der Patella in Bezug auf die Trochlea und eine Instabilität der TEP durch Varus- und Valgusstress mit Blick auf das Inlay evaluiert werden.

Nach der Aufarbeitung der Proben kann ein differenzierter Plan für eine mögliche Revisionsoperation mit verbesserter Risikoabschätzung entwickelt werden. Die histologische Untersuchung gibt neben der Analyse der Synovialzellen bezüglich der β‑Catenin-Expression einen Eindruck über das Vorliegen anderer pathologischer Prozesse (Infektion, Abriebpartikel; s. Artikel zur Diagnostik von Krenn und Krenn in dieser Ausgabe). Bei fehlendem Gelenkerguss kann die Operation als arthroskopische Arthrolyse fortgesetzt werden.

### Arthroskopische Arthrolyse

Die arthroskopische Arthrolyse wird für die Behandlung einer Bewegungseinschränkung sowohl am Schultergelenk („frozen shoulder“ [11]) als auch am Kniegelenk bei Arthrofibrose [12] und am Sprunggelenk (arthrotisch induzierte Bewegungseinschränkung [13]) empfohlen. Die Zugangsmorbidität wird dadurch verringert, und durch die Verwendung von Hochfrequenzelektroden ist eine gleichzeitige Blutstillung möglich. Auch bei einer arthroskopischen Arthrolyse kommt es zu Gewebsnekrosen mit Initiierung einer Inflammation. Diese sollte analog zu einer konservativen Arthrofibrosetherapie durch die Verbesserung der reparativen Prozesse [6, 26] behandelt werden.

Der Patient sollte für die Arthrolyse so gelagert werden, dass alle Bereiche, in denen Narben durchtrennt werden sollen, gut zugänglich sind. Dies kann durch eine Standardarthroskopielagerung oder durch die Lagerung mithilfe eines elektrischen Beinhalters umgesetzt werden. Die Blutsperre wird im eigenen Vorgehen nur vorgelegt, um eine Nachblutung kontrollieren zu können und die bestmögliche Blutstillung zu erreichen.

**Figure 2:**
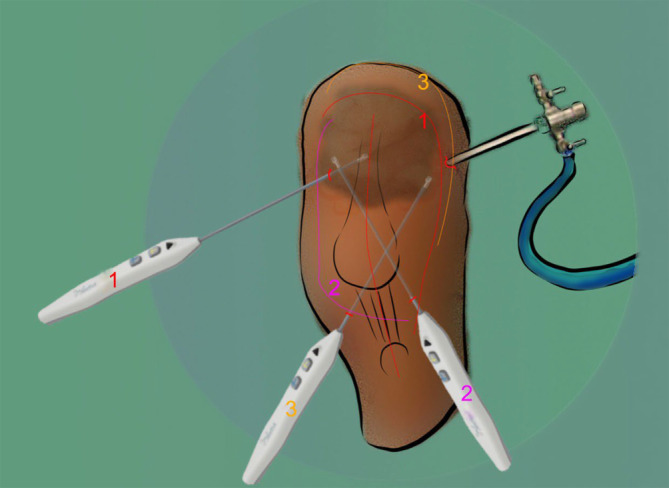

Bei einliegender TEP beginnt die Arthrolyse über 2 Zugänge im oberen Recessus, einer wird für die Optik, der andere als Arbeitskanal angelegt (Abb. 2). Ausgehend von der Erweiterung des Blickfelds im oberen Recessus wird die Arthrolyse schrittweise auf das gesamte ventrale Kniegelenk ausgedehnt, ohne die Oberfläche der TEP zu beschädigen. Die Arthrolyse des ventralen Kniegelenks erfolgt minimal-invasiv über 3 bis 4 Portale. Die fibrotische Bindegewebsschicht wird in den Positionen 1 bis 3 mithilfe einer Hochfrequenzablationselektrode (z. B. Fa. Arthrex, Naples, FL, USA) durchtrennt, bis die Patella wieder an Beweglichkeit gewinnt und eine Flexion gegen die Schwerkraft von mindestens 90 Grad möglich wird. Blutungen aus dem Bindegewebe, insbesondere aus der Muskulatur, werden unterbunden bzw. vermieden.

Über das anteromediale Portal kann auch Interkondylärgewebe entfernt oder durchtrennt werden. Im Fall eines ultrakongruenten Inlays ist die Durchtrennung des hinteren Kreuzbands nach eigener Erfahrung möglich. Die Arthrolyse kann bei bestehendem Streckdefizit auch nach dorsal fortgesetzt werden. Hierzu ist ein dorsomediales Portal erforderlich. Die Einlage einer Redon-Drainage ist nicht zwangsläufig notwendig. Periartikuläre Infiltrationen mit Ropivacain sind perioperativ schmerzmindernd (Level of evidence: I) [14]. Die intraartikuläre Injektion von Steroiden ist hingegen bei einer Arthrofibrose unzureichend untersucht und wirkt in vitro proliferationsfördernd [15].

#### Mobilisierung des Streckapparats

Eine Durchtrennung des Streckapparats abseits der Tuberositas tibiae stellt zwar die Flexion des Kniegelenks wieder her, verursacht jedoch aufgrund des Verlusts der muskulären Kontrolle des Kniegelenks eine Gehunfähigkeit. Diese Technik („quadriceps snip“ [16]) geht mit einer erheblichen Schwächung des Streckapparats einher [17] und wird daher nicht empfohlen. Bei Patienten, die mit einer Durchtrennung der Quadrizepssehne behandelt wurden, ist neben der Arthrolyse eine Rekonstruktion des Streckapparats notwendig, um die Gehfähigkeit wiederherzustellen. Die Limitierung der Beugung stellt für die Rehabilitation eine besondere Herausforderung dar. Es muss dann zunächst der Streckapparat rekonstruiert und im zweiten Schritt die Beugefähigkeit wiederhergestellt werden.

Die für spastische Kinder entwickelte Mobilisation des gesamten Streckapparats nach Judet [8, 18] hat zwar insbesondere für posttraumatische Zustände mit ausgedehnter Narbenbildung der Quadrizepsmuskulatur bis hin zum proximalen Oberschenkel eine Bedeutung, diese ist jedoch bei einer Arthrofibrose des Kniegelenks nach Band‑, Knorpelrekonstruktion oder Implantation einer Knie-TEP seltenst erforderlich.

**Figure 3:**
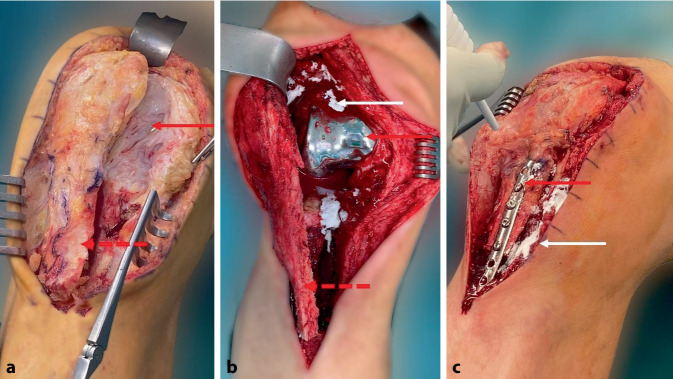

Der Versatz der Tuberositas tibiae ist in ausgeprägten Fällen in der eigenen Einschätzung die sinnvollere Alternative, da unmittelbar nach der Operation wieder eine aktive Streckfähigkeit des Gelenks möglich ist. Die knöcherne Schuppe sollte vor dem Hintergrund der Kranialisierung mit mindestens 7 cm dimensioniert und die Verankerung v. a. bei einer Langschaft-TEP mit „sleeves“ gut geplant werden (Abb. 3). Die Tuberositas kann in der Wunschposition zunächst mithilfe von Kirschner-Drähten fixiert und später mit einer Krallendrittelrohrplatte fixiert werden. Es sollte auf eine bestmögliche Restdurchblutung von Tibia und Tuberositas geachtet werden, um eine schnelle Einheilung zu ermöglichen. Bei einer erheblichen Ausdünnung der Weichteile kann eine Verbesserung der lokalen Perfusion durch einen Gastroknemius-Lappen-Transfer erreicht werden.

**Figure 4:**
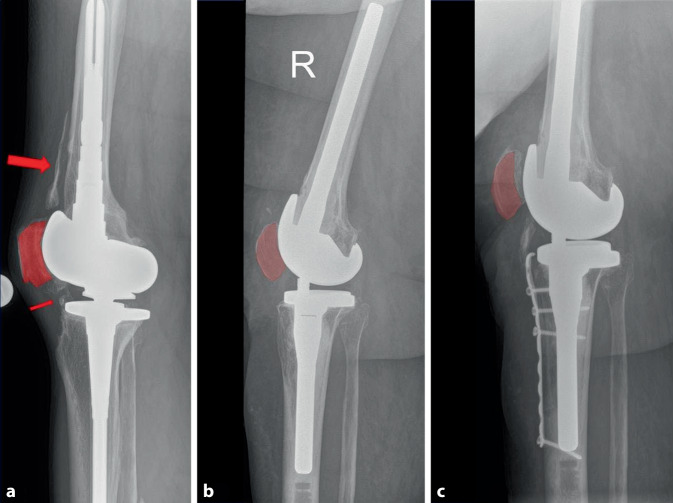

Die Beübung erfolgt aus der Flexion heraus in die Extension. Eine Vollbelastung ohne Streckung gegen Widerstand ist unmittelbar postoperativ möglich (Abb. 4 und 5).

**Figure 5:**
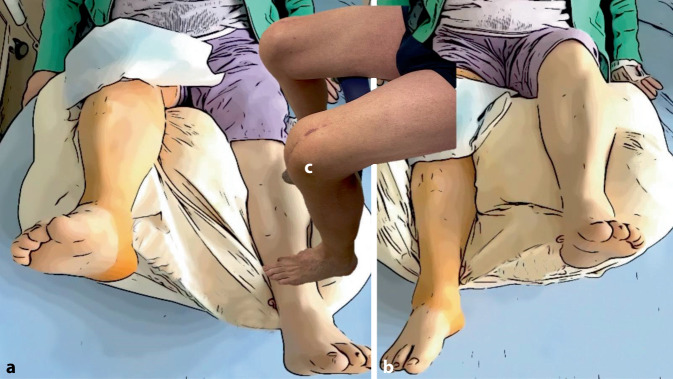

#### Wechsel des Implantats

**Figure 6:**
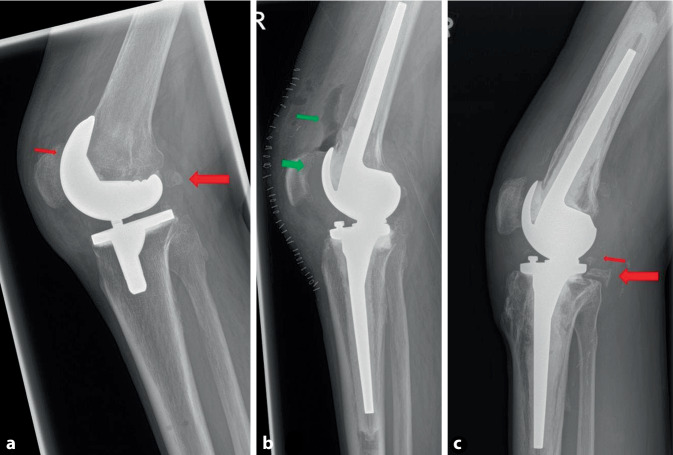

Ein Implantatwechsel wird zwar in einigen Studien als Mittel der Wahl zur Behandlung der Arthrofibrose nach einer Knie-TEP angegeben [19, 20], ist jedoch immer mit einem Knochensubstanzverlust vergesellschaftet und hat im Vergleich zu den oben genannten Optionen das höchste Risiko eines Rezidivs. Die Standzeiten einer Revisions-TEP sind im Endoprothesenregister gegenüber dem Primärimplantat deutlich reduziert [21]. Vor diesem Hintergrund empfehlen die Autoren des vorliegenden Beitrags den Wechsel der TEP nur in den Fällen, in denen nach erfolgter Arthrolyse eine Instabilität der TEP besteht, die nicht mithilfe einer Inlay-Erhöhung oder eines Teilwechsels behoben werden kann. Bei häufig eingeschränkter Knochensubstanz und Narbenbildung des Streckapparats bietet sich eine TEP mit schlanker Trochlea-Geometrie an. Hier wird zwar die Kraft im Streckapparat reduziert, jedoch ist die Beugefähigkeit auch bei fehlender Elastizität des Streckapparats verbessert. Für den TEP-Wechsel ist häufig eine Tuberositas-Osteotomie erforderlich (Abb. 6). Daher wird der TEP-Wechsel im eigenen Vorgehen nur bei TEP-Lockerung oder möglicher Instabilität aufgeklärt und dann bei Bestätigung der Instabilität und/oder Lockerung nach Arthrolyse durchgeführt (Abb. 6).

##### Merke.

Entgegen publizierter Daten [19, 20] wird vor dem Hintergrund der deutlich reduzierten Standzeiten [21], insbesondere bei jungen Patienten, ein Implantatwechsel bei bestehender Arthrofibrose nach einer primären TEP nur im Ausnahmefall empfohlen.

### Verbesserung des Behandlungserfolgs

Die Verbesserung der Wundheilung kann durch die folgenden Maßnahmen erreicht werden:

- Erhöhung der körperlichen Fitness vor der Revisionsoperation (Beseitigung einer schweren Adipositas), Schmerztherapie, Verbesserung der Muskelfunktion trotz Einschränkung der Beuge- und Streckfähigkeit.
- Klärung der Begleitfaktoren, Behandlung einer depressiven Verstimmung, Reduktion von emotionalen Stressoren, Arbeitsplatzverbesserung oder Rentenbegehren.
- Perioperative Schmerztherapie: Der Einsatz eines Schmerzkatheters ist auch hier umstritten, eine Verletzungsgefahr durch mögliche Einschränkung der motorischen Funktion des Streckapparats ist problematisch. Schmerzhaftes Beüben erscheint in jedem Fall kontraproduktiv und ist häufig traumatisierend; ein Biofeedback-gesteuertes Training unter Beachtung der individuellen Schmerzgrenzen hat sich bewährt (Abb. 5).
- Organisation einer geeigneten Rehamaßnahme, diese kann je nach Selbstständigkeit des Patienten häufig ambulant erfolgen, da die Behandlung in jedem Fall mehrere Monate benötigt.
- Medikamentöse Unterstützung: Während die intraartikuläre Applikation von Dexamethason in vitro proliferationsfördernd auf Fibroblasten wirkt [15], reduziert die systemische Gabe von Prednisolon in absteigender Dosierung die Inflammation des Gelenks. Es sollte auf die Gabe eines Protonenpumpenhemmers geachtet werden. Die Verabreichung von Vitamin D [22] und Angiotensin-II-Rezeptor-Antagonisten [23] wurde bei der Behandlung der Lungenfibrose erprobt und kann in Zukunft eine interessante Option der medikamentösen Therapie der Arthrofibrose darstellen.

#### Merke.

- Die Arthrolyse des Kniegelenks verbessert selten die Schmerzhaftigkeit und ist insbesondere bei einer schmerzarmen Bewegungseinschränkung erfolgreich.
- Bei schmerzhafter Bewegungseinschränkung sollten immer eine begleitende Schmerztherapie und eine psychosomatische Unterstützung sowie die Klärung eines Rentenbegehrens erfolgen.

## Fazit für die Praxis

- Die Arthrofibrose ist eine seltene, aber schwere Komplikation nach einer Gelenkoperation und -verletzung.
- Bei früher Diagnose und stadiengerechter Behandlung können Folgeoperationen und Narkosemobilisierung häufig vermieden werden.
- Eine frühe Diagnose und stadiengerechte Behandlung machen Folgeoperationen häufig unnötig.
- Im späten Stadium der Erkrankung ist eine konservative Behandlung in der überwiegenden Mehrzahl der Fälle nicht mehr mit einer Verbesserung der Beweglichkeit des Gelenks verbunden. Eine chirurgische Therapie muss gut vorbereitet und mit einer antifibrotischen Nachbehandlung geplant werden, um die Rezidivquote zu senken.
